# Supplementary material for: The Association Between Serum Drug Concentration and a Flare in Rheumatoid Arthritis Patients Tapering TNF Inhibitors
Source: Pharmaceuticals (Basel). 2025 Oct 8;18(10):1506. doi: 10.3390/ph18101506 (PMC12566885; doi:10.3390/ph18101506)
Supplement: Supplementary file 1 [file pharmaceuticals-18-01506-s001.zip › pharmaceuticals-3841129-supplementary.pdf]

Supplementary materials

Supplementary Figure S1. Flowchart showing patients disposition in the TARA trial and patients selected for the current analysis.

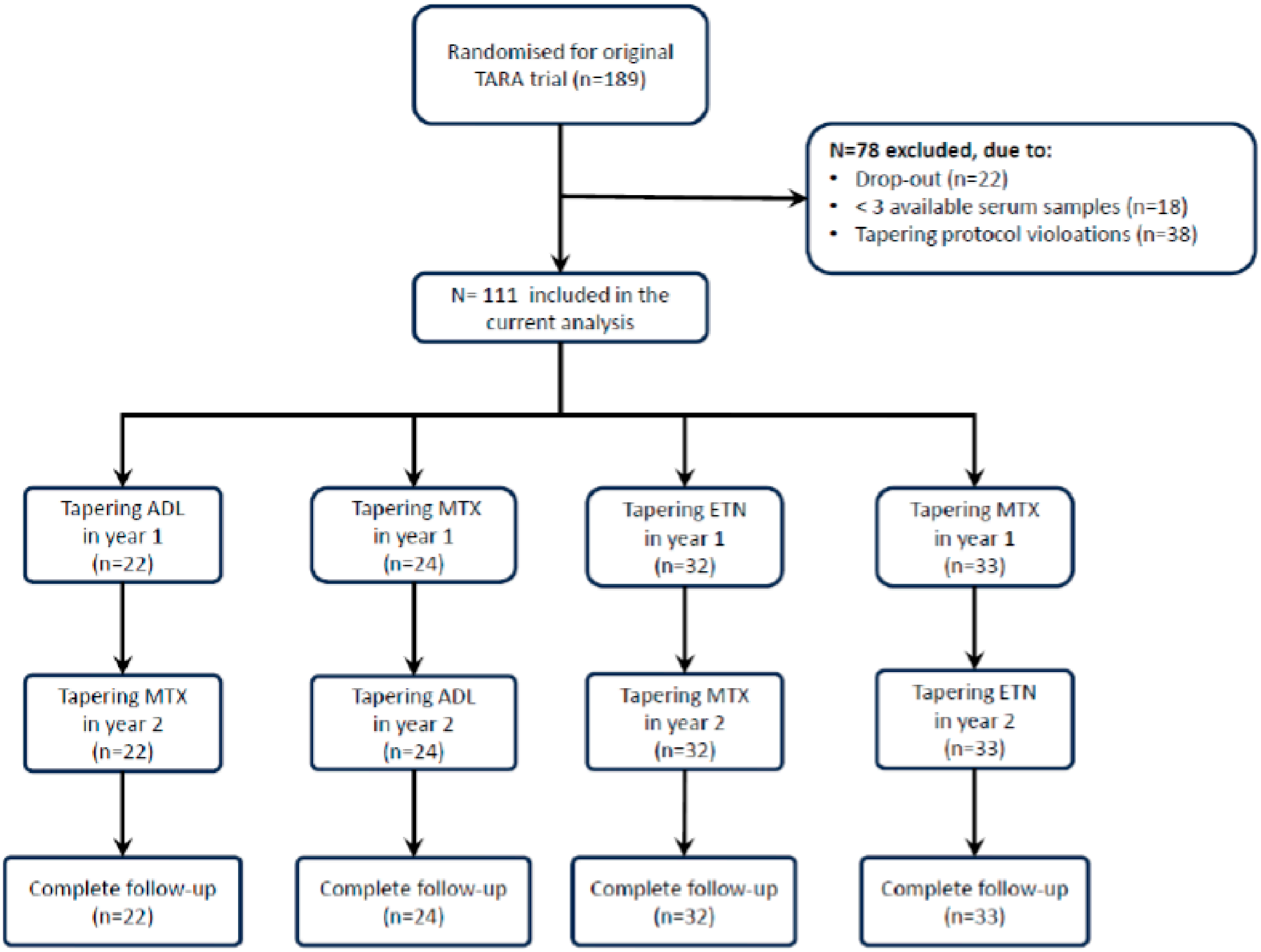

Table S1 Characteristics between included and excluded patients

Characteristics	*Included RA patients (N=111)*	*Excluded RA patients (N=78)*
Demographic		
• *Age (years), mean (sd)*	57 (11.3)	56 (13.7)
• *BMI, median (IQR)*	26 (24 – 29)	27 (24 – 31)
• *Sex, female, n (%)*	69 (62)	60 (73)
• *Disease duration (years), median (IQR)*	5.7 (3.9-8.5)	6.7 (4.9-9.0)
Clinical		
• *RF positive, n (%)*	56 (50)	53 (65)
• *ACPA positive, n (%)*	70 (63)	58 (71)
• *DAS44, mean (sd)*	1.0 (0.49)	1.1 (0.59)
• *ESR, median (IQR)*	8 (3-15)	8 (2-15)
• *CRP, median (IQR)*	2 (1-5)	3 (1-6)
• *Experienced a flare during entire follow-up, n (%)*	74 (67)	42 (51)
• *MTX dose in mg/week at start, median (IQR)*	20 (15 – 25)	20 (10 – 25)

Supplementary Text S1: Protocol Tara Trial

Patient Population:

The study included participants from the TApering strategies in Rheumatoid Arthritis (TARA) trial (NTR2754), spanning from September 2011 to July 2016. This multicenter, single-blinded randomized trial was conducted across 12 rheumatology centers in the southwestern part of the Netherlands. Adult rheumatoid arthritis (RA) patients with a well-controlled disease, defined as a Disease Activity Score (DAS) ≤2.4 and a swollen joint count (SJC) ≤1 at two consecutive time points within a 3-month interval, using a combination of conventional synthetic disease-modifying antirheumatic drugs (csDMARDs) and TNF inhibitors, were eligible for inclusion. The study protocol received approval from the medical ethics committees of each participating center, and all patients provided written informed consent before enrollment.

Tapering Schedule:

Participants were randomized into tapering their csDMARD in the first year followed by tapering the TNF inhibitor in the second year, or vice versa. The tapering process for both medications involved gradual reduction in three steps, with adjustments made every 3 months as long as disease control was maintained (see figure 1). The MTX dose was first halved, then quartered and thereafter it was stopped. Tapering of TNF inhibitors was commenced by first doubling the dose interval, followed by halving the dose, and finally it was stopped. Glucocorticoids (GCs) were not allowed at the start of the study, but there were no restrictions on the use of non-steroidal anti-inflammatory drugs or intra-articular GC injections.

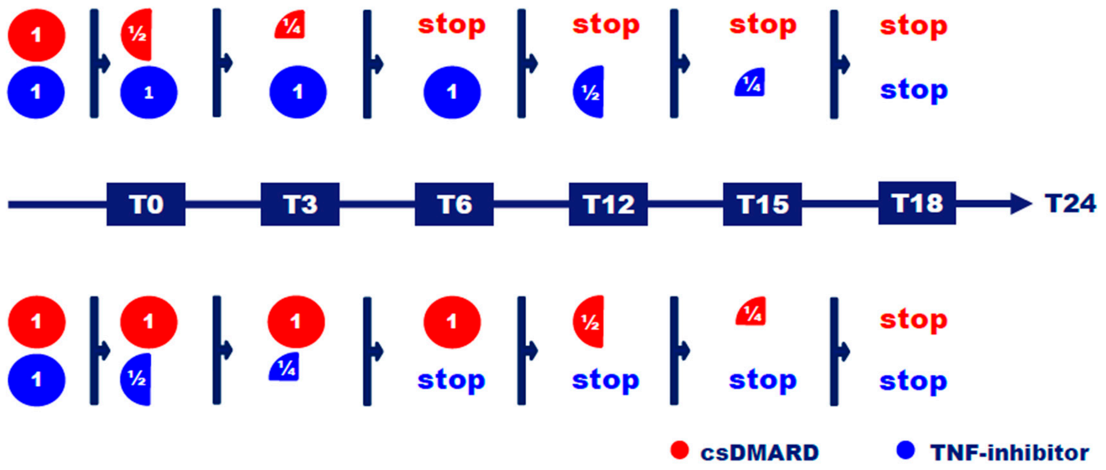

Figure S2. Tapering Schedule of csDMARD and TNF-inhibiter in TARA trial.

Randomization and Blinding:

Patients were randomized using minimization randomization stratified for center. Trained research nurses, blinded to the assigned tapering arm, assessed patients and calculated the DAS.

Assessments and Outcomes:

Patients underwent assessments at baseline and every 3 months thereafter, including measurement of DAS, medication usage, and completion of self-reported questionnaires. Hand and foot radiographs were obtained at baseline and after 1 and 2 years of follow-up. Adverse events (AEs), both regular and serious, were monitored throughout the study. The primary outcome was the proportion of patients experiencing a disease flare, defined as a DAS >2.4 and/or SJC >1, over the 2-year follow-up period.

Supplementary Figure S3: Adalimumab measurement

The adalimumab level determination is an ELISA with indirect coating of TNF-α, which operates according to the following principle (see figure below): The wells of a microtiter plate are coated with anti-TNF-α and then incubated with recombinant human TNF-α (in HPE, to prevent non-antigen-specific antibody binding). After incubation with patient serum, any present adalimumab can bind to the recombinant human TNF-α. This adalimumab can be detected with biotinylated rabbit anti-adalimumab F(ab')2 antibodies. By adding streptavidin coupled to an enzyme (HRP), the conversion of a colorless substrate (TMB) to a colored product is catalyzed. The concentration of the colored end product can be quantified with a spectrophotometer and is theoretically proportional to the concentration of adalimumab in the patient serum.

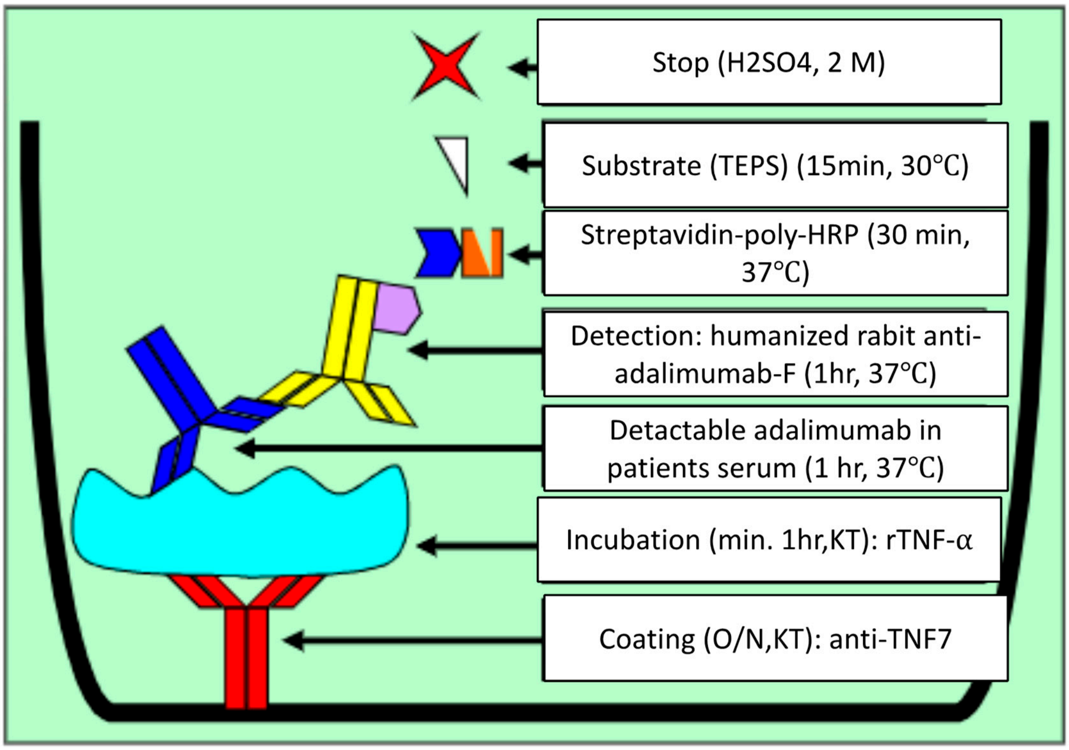

Supplementary Figure S4: Etanercept Measurement

The etanercept level determination is an ELISA with indirect coating of TNF-α, which operates according to the following principle (see figure below): The wells of a microtiter plate are coated with anti-TNF5 and then incubated with recombinant human TNF-α (in HPE, to prevent non-antigen-specific antibody binding). After incubation with patient serum, any present etanercept can bind to the recombinant human TNF-α. Etanercept can then be detected with biotinylated rabbit anti-etanercept antibodies. By adding streptavidin coupled to an enzyme (HRP), the conversion of a colorless substrate (TMB) to a colored product is catalyzed. The concentration of the colored end product can be quantified with a spectrophotometer and is theoretically proportional to the concentration of etanercept in the patient serum.

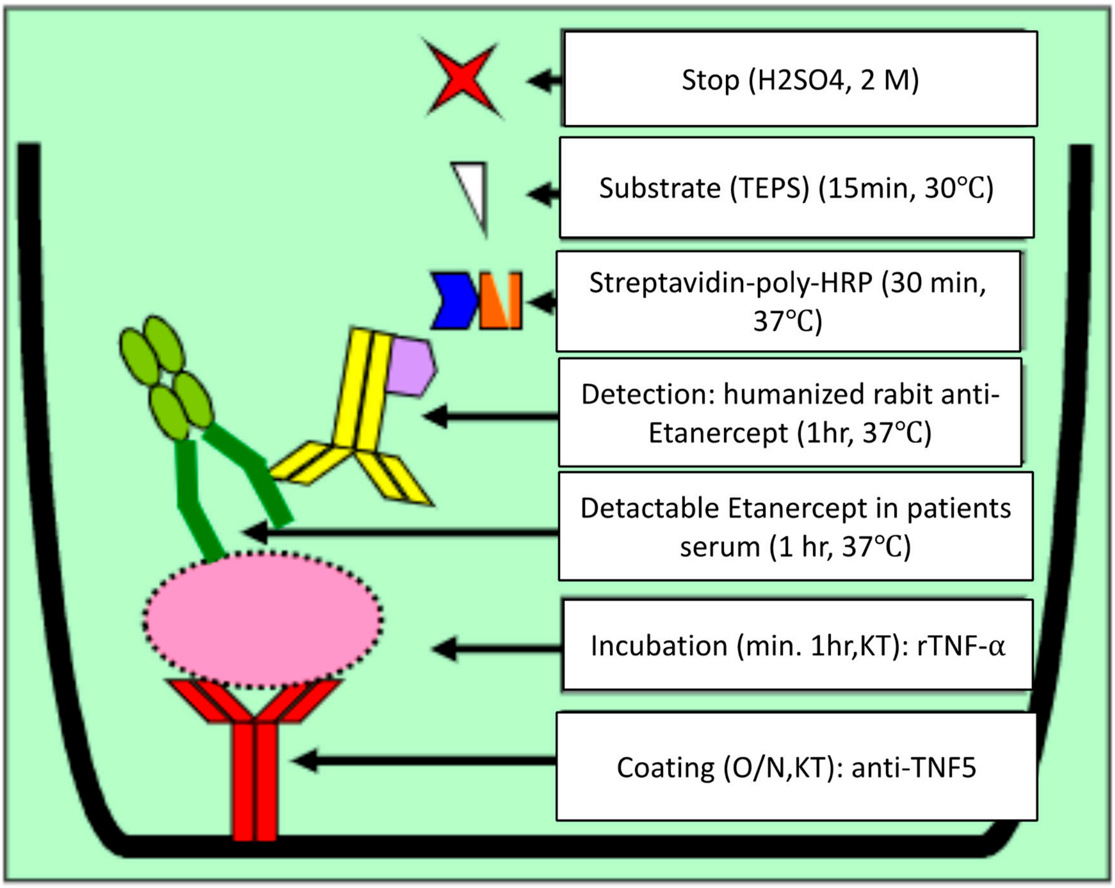

Supplementary Figure S5: Anti-drug antibody measurement

The ALFIA method relies on the fluorescence of europium. In this technique, the detection of anti-adalimumab antibodies (ADAs) on the bead surface has been done by using europium. Detection can occur directly with europium-labeled F(ab')2 adalimumab or indirectly with biotinylated F(ab')2 adalimumab followed by europium-labeled streptavidin (Strep-Eu). Protein labeling with europium is facilitated by a chelating agent, and europium elution from the beads is achieved with an excess of this chelator. The concentration of free europium chelate complex is then measured using time-resolved fluorescence spectrophotometry.

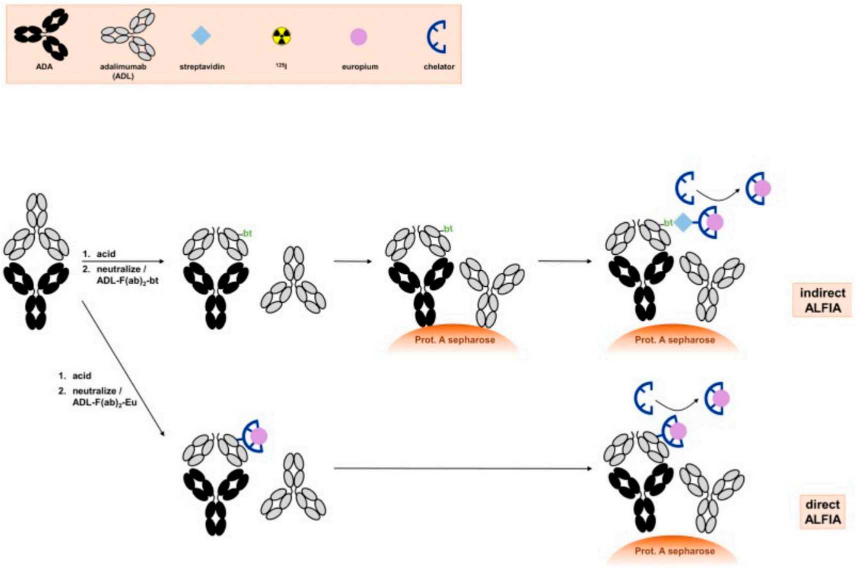

Supplementary Figure S6: Drug survival analysis in RA patients tapering ADL and ETN.

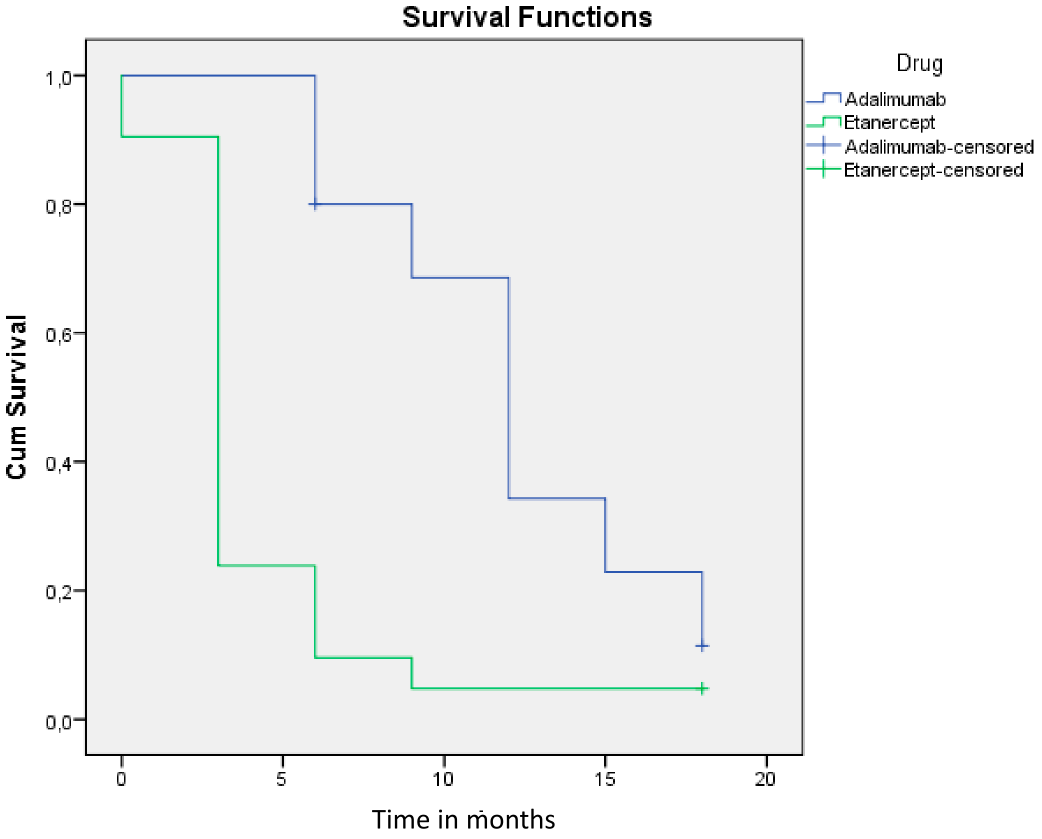

Each line demonstrates how long it takes before the ADL and ETN drug levels are unmeasurable in the blood circulation after cessation in RA patients with a well-controlled disease (drug elimination).

Abbreviations: ADL, Adalimumab; ETN, Etanercept; RA, Rheumatoid Arthritis

Supplementary Figure S7: Individual serum concentration of ADL in well-controlled RA patients with different tapering strategies.

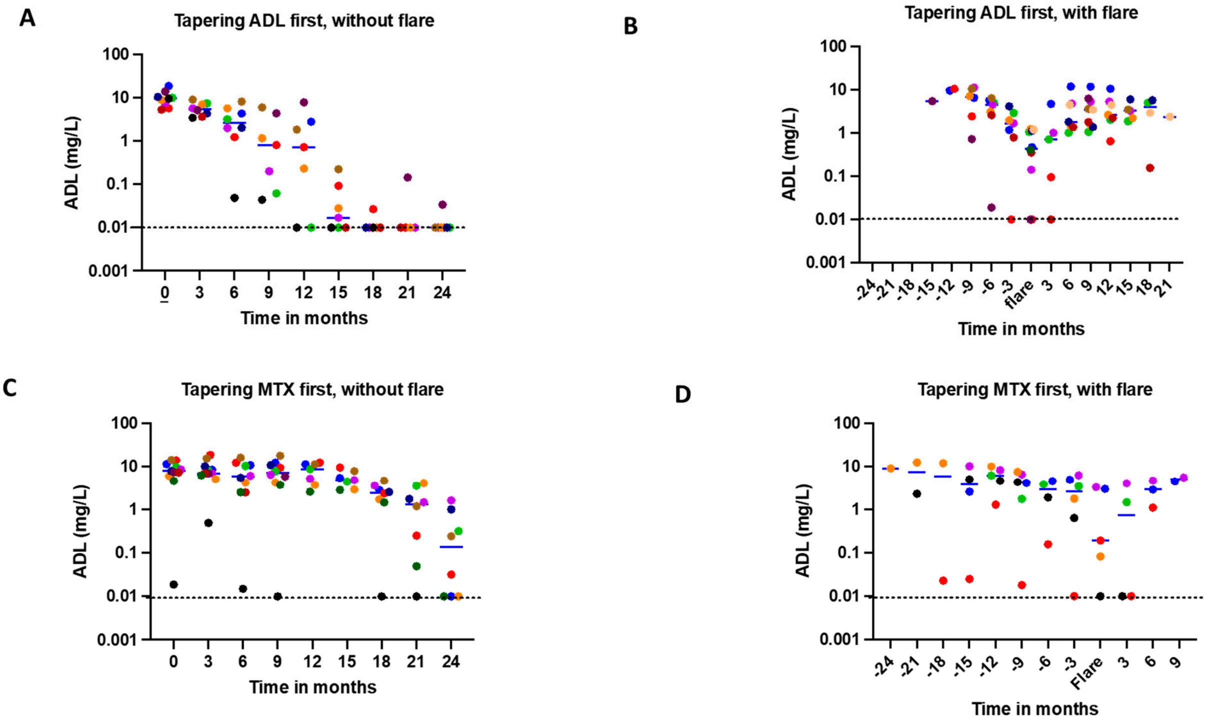

Each dot represents an ADL drug level measurement per individual RA patient. Measurements within each individual patient have the same color dot. Supplementary figure S6A (patients without a flare) and S6B (patients with a flare) show the ADL drug levels of RA patients who tapered the ADL in the first year and MTX in the second year. Supplementary figure S6C (without a flare) and S6D (with flare) again show ADL drug levels, but then for RA patients who tapered the MTX in the first year and ADL in the second year. The dotted line shows the detection limit of the ADL assay of 0.01 mg/L.

Abbreviations: ADL, Adalimumab; MTX, methotrexate; RA, Rheumatoid Arthritis

Supplementary Figure S8: Individual serum concentration of ETN in well-controlled RA patients with different tapering strategies.

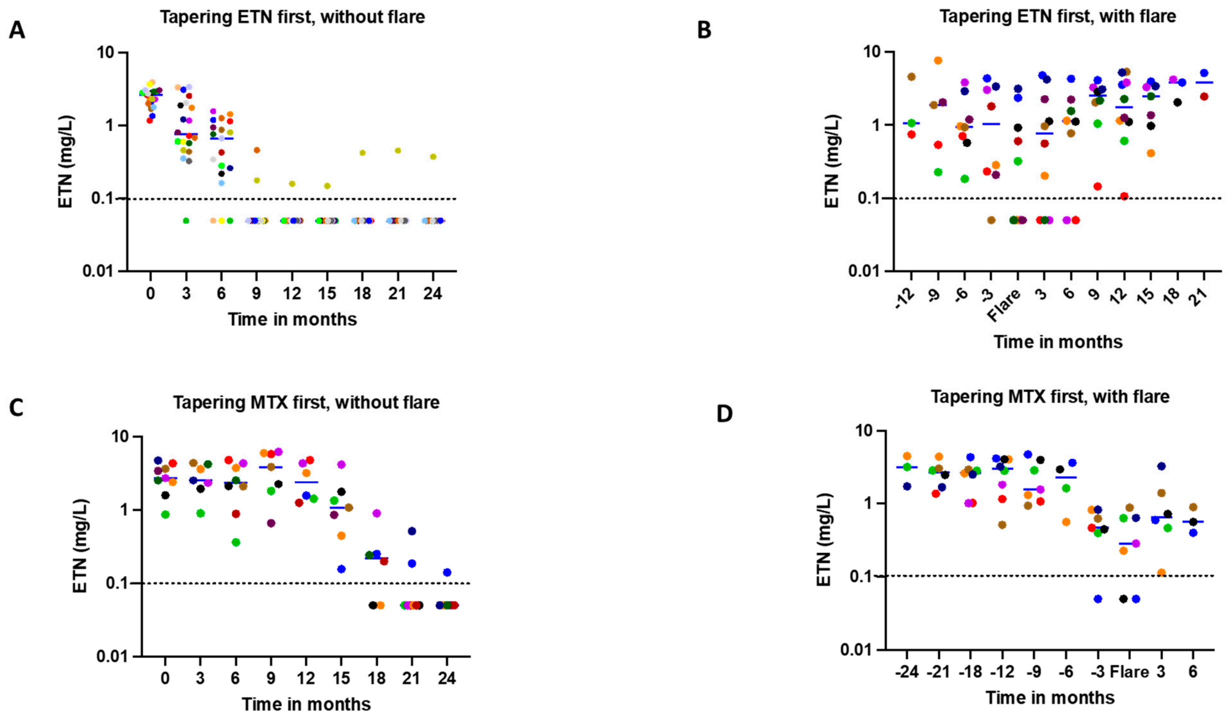

Each dot represents an ETN drug level measurement per individual RA patient. Measurements within each individual patient have the same color dot. Supplementary figure S7A (patients without a flare) and S7B (patients with a flare) show the ETN drug levels of RA patients who tapered the ETN in the first year and MTX in the second year. Supplementary figure S7C (without a flare) and S7D (with a flare) again show ETN drug levels, but then for RA patients who tapered the MTX in the first year and ETN in the second year. The dotted line shows the detection limit of the ETN assay of 0.1 mg/L.

Abbreviations: ETN, Etanercept; MTX, methotrexate; RA, Rheumatoid Arthritis

Supplementary Figure S9: Individual serum concentration of ADL and ETN in well-controlled RA patients experiencing a flare during tapering and restarting the treatment.

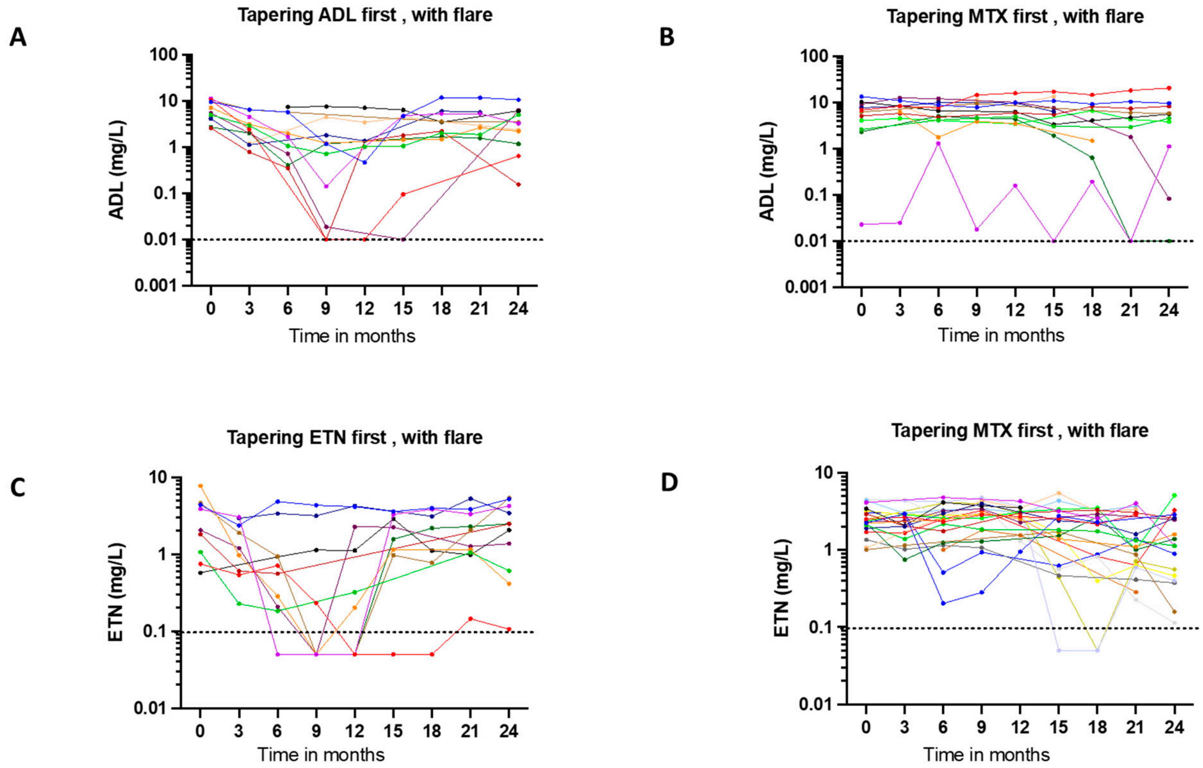

Each point on the graph represents a measurement of ETN or ADL drug levels for an individual RA patient who underwent tapering and experienced a disease flare. Measurements for each patient are indicated by dots of the same color and are connected over time by lines. Supplementary Figure S8A (tapering ADL first) and S8B (tapering MTX first) illustrate the ADL drug concentrations in RA patients who encountered a flare during tapering and subsequently resumed treatment. Supplementary Figure S8C (tapering ETN first) and S8D (tapering MTX first) depict ETN drug concentrations in patients experiencing a flare and restarting treatment over time. The dotted line shows the detection limit of the ADL and ETN assay of 0.01 and 0.1 mg/L, respectively.

Abbreviations: ADL, Adalimumab; ETN, Etanercept; MTX, methotrexate; RA, Rheumatoid Arthritis
